# Maternal prenatal cortisol predicts infant negative emotionality in a sex-dependent manner

**DOI:** 10.1016/j.physbeh.2017.03.017

**Published:** 2017-06-01

**Authors:** Elizabeth C. Braithwaite, Andrew Pickles, Helen Sharp, Vivette Glover, Kieran J. O'Donnell, Florin Tibu, Jonathan Hill

**Affiliations:** a School of Psychology and Clinical Language Sciences, University of Reading, Reading, UK; b Department of Experimental Psychology, University of Oxford, Oxford, UK; c Institute of Psychiatry, Psychology and Neuroscience, Kings College London, London, UK; d Department of Psychological Sciences, Institute of Psychology, Health and Society, Liverpool, UK; e Institute of Reproductive and Developmental Biology, Imperial College London, London, UK; f Douglas Hospital Research Centre, McGill University, Montreal, Canada; g Canadian Institute For Advanced Research, Child and Brain Development Program, Ontario, Canada; h Institute of Child Development, Bucharest, Romania

**Keywords:** Prenatal stress, Cortisol, Fetal programming, Sex differences, Infant behaviour

## Abstract

**Objective:**

Prenatal stress influences fetal developmental trajectories, which may implicate glucocorticoid mechanisms. There is also emerging evidence that effects of prenatal stress on offspring development are sex-dependent. However, little is known about the prospective relationship between maternal prenatal cortisol levels and infant behaviour, and whether it may be different in male and female infants. We sought to address this question using data from a prospective longitudinal cohort, stratified by risk.

**Method:**

The Wirral Child Health and Development Study (WCHADS) cohort (n = 1233) included a stratified random sub-sample (n = 216) who provided maternal saliva samples, assayed for cortisol, at home over two days at 32 weeks of pregnancy (on waking, 30-min post-waking and during the evening) and a measure of infant negative emotionality from the Neonatal Behavioural Assessment Scale (NBAS) at five weeks-of-age. General population estimates of associations among measures were obtained using inverse probability weights.

**Results:**

Maternal prenatal cortisol sampled on waking predicted infant negative emotionality in a sex-dependent manner (interaction term, p = 0.005); female infants exposed to high levels of prenatal cortisol were more negative (Beta = 0.440, p = 0.042), whereas male infants were less negative (Beta = − 0.407, p = 0.045). There was no effect of the 30-min post-waking measure or evening cortisol.

**Discussion:**

Our findings add to an emerging body of work that has highlighted sex differences in fetal programming, whereby females become more reactive following prenatal stress, and males less reactive. A more complete understanding of sex-specific developmental trajectories in the context of prenatal stress is essential for the development of targeted prevention strategies.

## Highlights

• Prenatal cortisol was associated with infant negative emotionality in a sex dependent manner.
• Females exposed to high prenatal cortisol were more negatively emotional, but males were less negatively emotional.
• There may therefore be sex differences in fetal programming mechanisms.
• There are implications for understanding sex differences in developmental psychopathology.

## 1 Introduction

Maternal prenatal depression and anxiety are associated with increased risk for adverse offspring outcomes, including: poor obstetric outcomes [1], [2], behavioural difficulties in childhood [3], and mental health disorders in adolescence [4], [5]. Notably, these effects appear to be independent of maternal postnatal mood [4], [6]. Although most studies are unable to rule out possible genetic confounds, results from an *in vitro* fertilisation study suggest that some effects of prenatal mood on fetal development are also independent of shared risk genes between mother and infant [7], highlighting potential *in utero* mechanisms as mediating processes.

Animal studies implicate alterations of the hypothalamic pituitary-adrenal (HPA) axis as a potential mediating mechanism in associations between prenatal stress and adverse offspring development [8], [9], however evidence from the human literature has been less consistent. The theory is that disturbances in maternal mood during pregnancy results in higher levels of circulating glucocorticoids, namely cortisol, which cross the placental barrier and alter fetal development. Indeed, a number of studies have demonstrated associations between heightened cortisol in pregnancy and adverse obstetric outcomes, including reduced birth weight and shortened gestational length [10], [11], [12], [13], [14], [15]. There have also been reports of associations between heightened maternal prenatal cortisol and negative emotionality and behaviour problems in children. In the largest study to date (N = 247), maternal salivary cortisol in late pregnancy was associated with maternal reports of more negative infant reactivity at 2 months of age [16]. Although this study assessed maternal salivary cortisol during each trimester, only cortisol in late pregnancy predicted infant reactivity. In a sample of 135 infants whose mothers underwent an amniocentesis during mid pregnancy, amniotic fluid cortisol was not associated with maternal reports of temperament at 3 months of age [14]. These two studies highlight that cortisol in late, but not mid pregnancy, may be a particularly important marker for offspring behaviour. Alternatively, in a study that assessed maternal reports of temperament and behavioural problems in older children (27 months), there was no association between maternal salivary cortisol sampled during each trimester and behavioural outcomes [17]. Thus, prenatal cortisol may be a salient predictor of early infant, but not childhood, behaviour. It is also plausible that variation in study methodologies, including different sample sizes, cortisol sampling procedures and measures of infant behaviour, could explain the disparate findings.

From the few existing studies, we can conclude that effects of maternal prenatal cortisol on early measures of infant behaviour are currently unclear. It is possible that associations between prenatal cortisol and infant behaviour may be sex-dependent. Indeed, sex differences in postnatal outcomes following exposure to prenatal risk have been described in the human and animal literature. For example, a number of studies have exposed pregnant dams to random daily stress, and have tested behaviour in the adult offspring. Many of these studies report elevated anxiety and depression-like behaviours in offspring exposed to maternal prenatal stress [18], including reduced exploration of the open arms of an elevated maze test [19] and increased length of immobility in the forced swim test, in females but not in males [20]. Further, adrenalectomy of the pregnant dams eliminated effects of prenatal stress on female offspring behaviour [19], consistent with a sex-dependent effect mediated *via* glucocorticoid mechanisms.

Accumulating evidence from the human literature also suggests that prenatal risks for offspring psychopathology may be sex-dependent. For example, prenatal risks have been reported to be associated with increased internalising symptoms in females but not males [5], [21], [22], and externalising behaviours in males but not females [23], [24]. Elevated cortisol in pregnancy has been shown to predict fearful temperament in girls at 2 months, and to predict pre-adolescent anxiety in girls. This effect was not seen in boys [25]. Elevated maternal cortisol predicts increased amgydala volume and more affective problems in girls but not boys [26]. Similarly, high prenatal anxiety has been related to a flattened diurnal cortisol profile and depressive symptoms in adolescent daughters [5]. Previous research from our group has found that maternal prenatal anxiety is associated with autonomic reactivity to challenge in a sex-dependent manner. High prenatal anxiety was associated with lower vagal withdrawal in response to the still face procedure at 29 weeks of age in boys, but higher vagal withdrawal in girls [27]. We also found that low birth weight was associated with vagal withdrawal in the same sex-dependent manner. This literature supports the emerging idea that processes underpinning fetal programming in the context of prenatal stress may be sex-dependent [25]; whereby females may become more reactive to challenge and anxious, and males become less reactive and more aggressive [25], [28].

However, more evidence is needed to support this idea, and key questions remain regarding effects of prenatal cortisol on early measures of infant behaviour. Critically, a more complete understanding of mechanisms by which prenatal stress impacts on development during early infancy is essential for the design of intervention and prevention strategies to avert the onset of later mental health difficulties. It is also critical to understand whether such pathways of effect may be different in male and female infants, so that intervention/prevention strategies may be targeted more effectively. Thus, the aims of the current study were to investigate effects of prenatal cortisol on early infant behaviour in a longitudinal cohort. Consistent with our previous vagal reactivity findings, we hypothesised that prenatal cortisol would predict infant irritability in a sex-dependent manner, whereby females exposed to high levels of prenatal cortisol become more behaviourally reactive (*i.e.* show more negative emotionality) and males show less negative emotionality.

## 2 Materials and methods

### 2.1 Design

Participants were members of the Wirral Child Health and Development Study (WCHADS), a prospective epidemiological longitudinal study of first-time mothers starting in pregnancy and with multiple follow-up assessments after birth. For some phases requiring more detailed and expensive measurement, data collection was restricted to a randomly drawn stratified sub-sample. The stratified design allows general population estimates of means and associations to be derived for measures from all phases. Recruitment of the cohort has been described in detail previously [29], [30]. Approval for the procedures was obtained from the Cheshire North and West Research Ethics Committee (UK).

### 2.2 Sample

The cohort consists of 1233 mothers, with a mean age at recruitment of 26.8 years (SD = 8.5, range = 18–51). Using the revised English Index of Multiple Deprivation (IMD) [31] based on data collected from the UK census in 2001, 41.8% fell in the most deprived UK quintile, consistent with high levels of deprivation in some parts of the Wirral. Only 48 women (3.9%) described themselves as other than White British.

The measures used in this report were obtained for the whole cohort from questionnaires at 20 weeks gestation and administrative records at birth, and the stratified sub-sample of mothers (n = 316) who provided interviews and saliva at 32 weeks gestation (mean 32.1, SD = 2.0) and additional questionnaires and the Neonatal Behavioural Assessment Scale (NBAS) (n = 282) of their infants at 5 weeks-of-age (mean 37 days, SD = 9).

### 2.3 Measures

#### 2.3.1 Maternal cortisol

At 32 weeks gestation, mothers collected saliva samples at home over two consecutive working days. Saliva was collected on waking, 30 min post-waking, and during the evening (approx. 12 h after waking (mean = 12h 10min, SD = 1 h 15 min)). Participants stored the samples in their freezer until a research assistant collected them 1–2 weeks later. Samples were then stored at − 20 °C before transportation to Imperial College London on dry ice for analysis. All samples were assayed for salivary cortisol using a commercially available immunoassay (Salimetrics, UK). Inter- and intra-assay variation were 7.9% and 8.9% respectively. Salivary assays were run in duplicate except for a small minority of cases with minimal volume (n = 3). The cortisol measures across the two days were highly correlated (waking: r = 0.485, p < 0.001, 30 minutes post-waking: r = 0.473, p = 0.001, and evening: r = 0.157, p = 0.02), and the mean over the two days of the waking awakening, 30-minutes post-waking and evening samples were used in analyses.

#### 2.3.2 Infant negative emotionality

The Neonatal Behavioural Assessment Scale (NBAS) was administered to the intensive sample 5 weeks after birth. The NBAS is a standardised measure designed to assess orienting, motor and emotional regulatory processed during the first weeks of life [32]. The administration and coding of the NBAS task within this cohort has been described in detail previously [29]. We used only the ‘irritability’ scale, which is a count of the number of occasions that the infant shows a change of state from calm, of at least 3 s, to fussing or crying in response to seven standard challenges. We have previously reported that infant negative emotionality, assessed in the same way, is predicted by an interaction between *MAOA-LPR* variants and life events in pregnancy [29]. The count of fuss/cry episodes provides a specific measure of reactivity paralleling maternal report and observational measures of temperament in later infancy, where responses to challenges such as restraint or unpredictable noises are assessed [33]. Three assessors were trained by Dr. Joanna Hawthorne, director of the UK Brazelton Centre. Pair-wise agreement (ICC) between independent ratings made from memory and video recordings on 220 infants ranged between 0.81 and 0.89.

#### 2.3.3 Stratification factor

For the sub-sampled phases of data collection different sampling fractions were used for those who scored low, intermediate and high on a maternal report of inter-partner psychological abuse. This variable was chosen for stratification in order to yield measurements on sufficient at-risk participants for analyses of psychopathological processes to be robust, but our weighted inferential analysis adjusted for the effects of this selection. Partner psychological abuse was assessed using a 20 binary-item questionnaire covering humiliating, demeaning or threatening utterances in the partner relationship during pregnancy and over the previous year [34], and has been described previously [29]. Participants first rated these items about their own behaviour towards their partner, and then about their partner's behaviour towards them. The stratification was based upon the highest of the partner to participant and participant to partner scores for each family.

#### 2.3.4 Confounders

We took account of the following confounders for which occasional missing values had been previously imputed; self-report prenatal Edinburgh Postnatal Depression Scale (EPDS) score (20-week) [35], maternal age, smoking in pregnancy and education, postcode based neighbourhood deprivation [31], infant birth weight by gestational age and obstetric risk index score [36] from hospital records. Additionally, we examined self-reported EPDS depression at 32 weeks of pregnancy and 5 weeks postnatally, State Anxiety Scale score [37] at 32-weeks of pregnancy, and hospital recorded 1-minute infant APGAR score.

### 2.4 Statistical analysis

The two-phase stratified sample design allows estimates to be reported for the general population by applying weights. Inverse probability weights were constructed that took account of the sample design stratification factor and variables associated with response and attrition: maternal age, depression and smoking in pregnancy, years of education, marital status and the deprivation index for the mother's home neighbourhood. The analysis method exactly compensates for differential selection and response, the stratified sampling and the weighting working as a pair, to balance out. Test statistics and confidence intervals for weighted means, and regression estimates were based on survey adjusted Wald tests (*t*-tests if single degrees of freedom (df) or F-tests (if multiple df)) using the robust ‘sandwich’ estimator of the parameter covariance matrix [38]. Models used for inference analysed the count of fuss/cry episodes using ordered logistic regression. Weights were calculated for each model separately, and a form of stabilized weight was used that removed weight variability associated with the conditioning covariates. To assist in interpretation, relationships estimated by simple unweighted regression have been displayed graphically.

Separate analyses examined predictions of irritability from the means across the two days for prenatal cortisol measure at waking, 30 min post-waking and during the evening. We estimated a series of models with infant negative emotionality as the outcome, introducing blocks of possible confounder variables that might explain or mediate associations with maternal cortisol. The first model included infant age as NBAS assessment and the stratification variable, the second model added prenatal cofounders and maternal pre- and postnatal affective symptoms, and the third model also included obstetric risk, 1-min APGAR score and fetal growth rate. For the associations with maternal prenatal cortisol itself, separate coefficients were estimated for male and female infants and each model was followed by a test of the equality of these male and female coefficients. The effects of confounders were also allowed to be sex specific. Analyses were undertaken in STATA 14 (2015).

## 3 Results

### 3.1 Demographic characteristics

Table 1 shows unweighted sample means and percentages split by gender for the prenatal cortisol and infant irritability variables, and the included confounders, stratification factor and measures associated with attrition for the sample with both maternal cortisol and negative emotionality measures. Male infants were exposed to similar levels of prenatal risks compared to females, showing only some excess smoking exposure in pregnancy. Mean differences in postnatal depression were also small.

### 3.2 Waking cortisol and infant negative emotionality

**Fig. 1:**
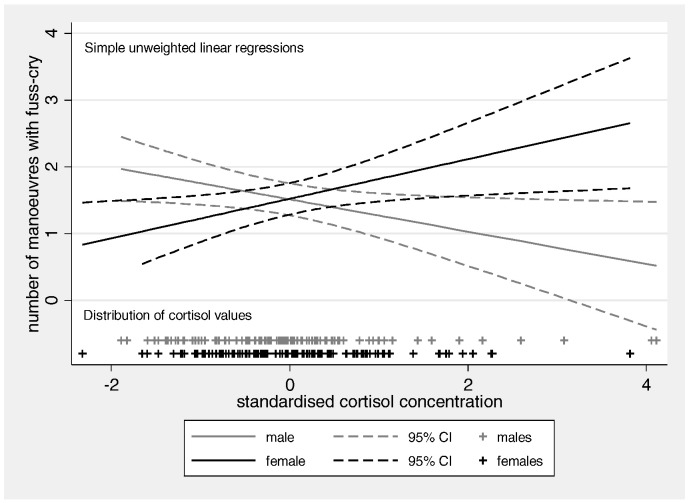
Maternal prenatal cortisol at 32 weeks of pregnancy and infant negative emotionality at five weeks-of-age on the NBAS by gender.

Table 2 Model 1 shows the coefficient estimates accounting for sample stratification and infant age at NBAS assessment and the discrete distribution of the negative emotionality measure. Significant effects of maternal cortisol for both boys and girls were evident and these effects were significantly different from each other. For illustration, Fig. 1 shows the simple unweighted linear regression effects of high prenatal maternal cortisol; showing the decreased negative emotionality for boys and the increased negative emotionality for girls. The addition of the prenatal confounders; maternal age, education, marital status, deprivation and smoking in pregnancy (of which none showed an association with reactivity, all p < 0.1), left the effects of cortisol unchanged (p = 0.045 for boys, p = 0.042 for girls), as did the addition of prenatal measures of affective symptoms (of which none were significant, all p < 0.1) (Table 2 Model 2). While the maternal postnatal EPDS score proved to be a significant predictor of reduced negative emotionality in girls (p = 0.037), the relationship of infant negative emotionality to maternal prenatal cortisol changed little, remaining significantly different for boys and girls. On addition of the final three confounders, obstetric risk, 1-min Apgar score and fetal growth rate, with the risk of over-controlling, the interaction remains strongly significant (p = 0.006) (not shown in table).

### 3.3 30-Min post-waking and evening cortisol, and infant irritability

For the maternal cortisol measures taken at 30 min post-waking and in the evening, there was no association with infant reactivity outcome in either boys or girls (data not shown).

## 4 Discussion

We conducted analysis of a longitudinal cohort, stratified by risk, to examine the prospective relationship between cortisol samples collected in the third trimester of pregnancy and infant negative emotionality assessed by observation at 5 weeks of age. Maternal cortisol samples collected at waking predicted infant negative emotionality in a sex-dependent manner: high waking cortisol was associated with increased irritability in females, and decreased irritability in males. However, maternal cortisol sampled at 30 min post-waking and during the evening did not predict negative emotionality.

Our findings support an emerging body of evidence that suggests that there may be sex differences in fetal programming mechanisms [25], [28]. These effects are perhaps best demonstrated in experimental animal models, where prenatal stress has been associated with a depressive/anxious phenotype [18], [20], [39], [40], [41], a persistent increase in reactivity of the hypothalamic pituitary-adrenal (HPA) axis [42] and also increased cardiovascular reactivity [43] in female offspring. Similarly, results from human studies have shown that following prenatal stress exposure, females present with a more fearful temperament [25], depressive/anxious symptoms [5], [44], and increased HPA [45], [46] and vagal [27] reactivity. In this context, the term ‘reactivity’ encompasses a broad construct, and implies that prenatal stress may prime a number of biological systems to function in a more active manner in response to challenge. Indeed, it is possible that increases in emotional, behavioural and physiological reactivity following prenatal stress exposure in females are underpinned by common biological mechanisms, and also may lead to common outcomes, such as a depressive/anxious phenotype in later life [25].

In addition to sex differences in fetal programming, our findings also have potential implications for sex differences in child and adolescent psychiatric disorders. Rates of neurodevelopmental and externalising disorders are higher in boys before puberty [47], and post-puberty there is a female predominance of mainly affective disorders [48], but the reasons for these sex differences are poorly understood. Elevated rates of externalising disorders in males are probably explained to a substantial degree by differential risk exposure [49]. For example, Moffitt et al. (2001) argue that higher levels of antisocial behaviour in males, as evidenced in the Dunedin cohort, are explained by a higher exposure of males to risks associated with antisocial behaviour [49]. However, there are at least two further possibilities that have received less attention. First, it is possible that risks for psychopathology are different in males and females, which may be particularly relevant to prenatal influences. For example, low birth weight and prenatal stress have been associated with adolescent depression in females, but not males [5], [21], [44]. Similarly, we have previously shown that prenatal anxiety predicts internalising symptoms in 2.5-year-old girls, but not boys, in the presence of low maternal stroking in the early postnatal period [30]. Our current findings are also consistent with this hypothesis, as we have shown that raised prenatal cortisol predicts increased irritability in girls, an early marker of negative emotionality associated with later poor social competence and psychopathology [50], [51]. A second alternative is that the risks for psychopathology may be the same for males and females, but the mechanisms leading to the onset of psychopathology are different. Evidence for this idea originated over 20 years ago, when Eisenberg et al., (1995) demonstrated that higher vagal tone was associated with improved social competence and emotion regulation in boys, but with poorer functioning in girls [52]. Recent studies have replicated this sex difference, whereby higher vagal tone or vagal withdrawal was associated with better functioning in boys and, critically, poorer functioning in girls [27], [53], [54]. It may that the opposite changes in vagal reactivity following prenatal adversity are on the causal pathway to different psychiatric outcomes for males and females. For example, greater vagal reactivity has been shown to predict more externalising behaviours in female, but not male, children [54]. The current study has demonstrated both an increase in negative emotionality in girls, and a decrease in negative emotionality in boys, following high prenatal cortisol exposure. We know that negative reactivity to frustrating events in infancy has been related to noncompliance [55], aggressive behaviour [56] and poor emotion regulation [57] in childhood. Thus, following prenatal cortisol exposure, females may be at increased risk of these outcomes, whereas reduced negative emotionality in males may represent reduced risk, or a protective mechanism. Alternatively, low reactivity may lead to certain forms of aggression in males, such as those associated with callous unemotional traits and low emotionality [58]. A clear direction for further research is to question whether the sex-specific changes in negative emotionality predict later childhood and adolescent psychiatric outcomes.

Another direction for future research is to further understand how the timing of prenatal cortisol measures may be relevant for infant outcomes. Existing research suggests that cortisol sampled in the third trimester of pregnancy may be particularly important for infant behavioural outcomes [14], [16], [17], and our findings are consistent with this. We also found that waking cortisol, but not cortisol at 30-min post-waking or during the evening, predicted negative emotionality. It is unclear why waking cortisol may be particularly relevant to infant behaviour, and existing studies do not provide information on time-of-day effects [14], [16], [17]. However, research focused on prenatal cortisol and obstetric outcomes, such as gestational age and birth weight, have highlighted that various indices of morning cortisol more strongly predict obstetric outcomes than measures taken throughout the day [11], [15], [59]. Our findings are consistent with the obstetric literature, but replication and further investigation is required.

This study has a number of strengths, notably the epidemiological sample recruited during pregnancy with a subsample stratified by psychosocial risk for more detailed assessment. This enables data from the time consuming observational measure of infant irritability derived from the subsample at 5 weeks of age to be weighted back to the general population. Our measures were assessed prospectively, and included a large number of relevant confounding variables. Limitations of the study include that participants' self-reported the timing of the cortisol sample collection, which could potentially be inaccurate and introduce error. We also had no information on time spent asleep before the first morning sample, or information on the time of the last meal/drink before each cortisol sample, which could affect the cortisol measurement [60], [61].

## 5 Conclusions

To conclude, this research has highlighted that in late pregnancy, maternal cortisol levels at waking predict infant behaviour in a sex-dependent manner, such that females have increased negative emotionality following high cortisol exposure, whereas males have decreased negative emotionality. When considering effects of prenatal stress on fetal developmental trajectories, there is accumulating evidence for sex differences in fetal programming mechanisms and the development of psychiatric disorders. Understanding the origins of such sex differences has implications for the design of targeted intervention and prevention strategies.

## Figures and Tables

**Table 1:** Cortisol, negative emotionality and potential confounding variables (unweighted random stratified sub-sample).

Variable type	Measure	Males				Females			
		N	Mean	SD	%	N	Mean	SD	%
Independent	Maternal waking cortisol (nmol/l)	101	12.25	5.25	–	115	12.58	4.60	–
	Maternal 30-min post-waking cortisol (nmol/l)	101	14.35	6.70	–	115	14.68	5.74	–
	Maternal evening cortisol (nmol/l)	101	4.33	3.03	–	115	3.92	2.25	–
Dependent	Count of NBAS fuss/cry maneuvers	101	1.51	1.33	–	115	1.51	1.33	–
Stratification Factor	Stratum - low psychological abuse	101	–	–	46	115	–	–	47
	Stratum - mid psychological abuse		–	–	14		–	–	13
	Stratum - high psychological abuse		–	–	41		–	–	40
Confounders	Infant age at NBAS (days)	101	36.56	7.99	–	115	36.23	7.70	
	Maternal age at consent (years) < 21 years	101	–	–	14	115	–	–	12
	22–30 years		–	–	53		–	–	56
	30–51 years		–	–	33		–	–	32
	Maternal education beyond age 18	101	–	–	61	115	–	–	68
	Most deprived quintile	101	–	–	40	115	–	–	42
	Maternal prenatal smoking (32 week)	101	–	–	22	115	–	–	11
	No partner	101	–	–	27	115	–	–	20
	Maternal prenatal depression (32 week)	98	8.23	4.80	–	115	7.90	4.41	–
	Maternal prenatal anxiety (32 week)	98	33.36	10.02	–	115	32.62	9.87	–
	Obstetric risk index	101	2.06	1.33	–	115	2.02	1.33	–
	Infant birth weight by gestational age (gms/day)	101	12.37	1.82	–	115	12.05	1.40	–
	Infant APGAR 1-min	98	9.04	1.36	–	110	8.95	1.58	–
	Maternal postnatal depression (5 weeks-of-age)	99	5.54	4.03	–	111	5.68	4.04	–

**Table 2:** Summary of ordered logistic regression models of prenatal waking cortisol and gender predicting infant irritability.

	Model 1 (n = 216)			Model 2 (n = 199)		
	Log-odds coefficient	CI's	P value	Log-odds coefficient	CI's	P value
Infant gender	− 0.281	− 2.540, 1.980	0.807	− 1.071	− 4.192,2.049	0.499
Age (weeks) at NBAS males	− 0.043	− 0.081, − 0.006	0.024	− 0.056	− 0.100, − 0.013	0.011
Age (weeks) at NBAS females	− 0.054	− 0.100, − 0.008	0.022	− 0.048	− 0.093, − 0.003	0.036
Prenatal waking cortisol males	− 0.440	− 0.801, − 0.079	0.002	− 0.407	− 0.805, − 0.008	0.045
Prenatal waking cortisol females	0.382	0.004, 0.761	0.048	0.440	0.016, 0.864	0.042
Test of equality of cortisol effects (cortisol × gender interaction)			0.003			0.005

Model 1: Additionally covaried for stratification.

Model 2: Additionally covaried for stratification, maternal age, education, marital status, deprivation, smoking in pregnancy, prenatal depression and anxiety (32 weeks), and postnatal depression (5 weeks).
